# Combining Transarterial Chemoembolisation With Reduced Deubiquitinase Activity Reduces Hepatocellular Carcinoma Progression

**DOI:** 10.1111/jcmm.71209

**Published:** 2026-06-15

**Authors:** Yun Tao, Wenhui Yu, Wenge Yang, Qinghua Wu, Jie Li

**Affiliations:** ^1^ Department of Intervention The Affiliated Hospital of Jiangnan University Wuxi China

**Keywords:** deubiquitination, hepatocellular carcinoma, PD‐L1, transarterial chemoembolisation, USP21

## Abstract

To investigate the interplay between ubiquitin‐specific protease 21 (USP21) and programmed death‐ligand 1 (PD‐L1) in the context of Transarterial chemoembolisation (TACE) treatment for hepatocellular carcinoma (HCC) and explore a novel combinatorial strategy to enhance therapeutic efficacy. USP21 was silenced in HCC cells and in two distinct mouse models: a diethylnitrosamine (DEN)‐induced orthotopic HCC model and a HepG2 subcutaneous xenograft model. The effects of USP21 knockdown, TACE and their combination on tumour growth, proliferation, invasion and apoptosis were evaluated. The regulation of PD‐L1 by USP21 was studied through co‐immunoprecipitation and deubiquitination assays. Rescue experiments were performed by overexpressing PD‐L1. Silencing USP21 attenuated HCC cell proliferation and invasion. Combining TACE with USP21 knockdown significantly enhanced antitumour effects compared to monotherapies. USP21 interacted with and deubiquitinated PD‐L1, stabilising its expression. Cycloheximide chase assays with densitometric quantification demonstrated that USP21 significantly prolonged the half‐life of PD‐L1 protein. Overexpression of PD‐L1 reversed the inhibitory effects of USP21 silencing and the enhanced therapeutic efficacy of the TACE/USP21 knockdown combination. USP21 stabilises PD‐L1 through deubiquitination, promoting HCC progression and immune evasion. Silencing USP21 enhances the therapeutic efficacy of TACE by modulating PD‐L1 expression, suggesting a promising combinatorial strategy for HCC treatment.

## 1 Introduction

Hepatocellular carcinoma (HCC) stands as the primary liver malignancy, driving a significant proportion of cancer‐related fatalities globally with an annual toll of nearly 850,000 new cases and 800,000 deaths [1]. The challenge of HCC comes from its aggressive nature, often being detected late and having limited treatment options [2]. While surgical resection and liver transplantation hold promise as curative measures for early‐stage HCC, the reality for most patients unfolds with advanced or unresectable disease, necessitating systemic interventions [3, 4]. Transarterial chemoembolisation (TACE) has become the standard treatment, involving the direct delivery of chemotherapy into the tumour's blood vessels, followed by arterial blockage to cause tumour cell death [5]. However, despite its widespread application, the efficacy of TACE remains compromised by inevitable tumour recurrence and metastasis, leading to suboptimal clinical outcomes, with a median overall survival of around 20 months [6, 7].

Central to the dynamics of tumour progression in HCC is programmed death‐ligand 1 (PD‐L1) [8], a pivotal immune checkpoint protein orchestrating the suppression of the antitumour immune response by binding to its receptor PD‐1 on T cells, thereby precipitating T cell exhaustion [9]. PD‐L1 overexpression in HCC is driven by various mechanisms, including genomic alterations, epigenetic modifications and post‐translational regulation. Oncogenic signalling pathways such as PI3K/AKT/mTOR and MAPK cascades, along with inflammatory cytokines like IFN‐γ and TNF‐α, contribute to its upregulation [10, 11]. Moreover, hypoxic conditions induced by TACE further exacerbate PD‐L1 expression [12], fostering an immunosuppressive tumour microenvironment that perpetuates immune evasion and augurs a bleak prognosis.

Concomitantly, ubiquitin‐specific protease 21 (USP21) emerges as a key player in HCC pathogenesis, exerting its influence through deubiquitination of target proteins to stabilise oncoproteins and drive tumour progression [13]. Upregulated USP21 enhances cell proliferation, migration, invasion and chemoresistance by stabilising critical oncogenic proteins [14]. Furthermore, USP21's involvement in regulating apoptosis, epithelial‐mesenchymal transition (EMT) and angiogenesis further fuels cancer progression and metastasis [15].

Despite the acknowledged roles of PD‐L1 and USP21 in HCC progression and therapy resistance, the interplay between these entities and its ramifications on TACE efficacy remain uncharted territory. In light of this, we hypothesised that silencing USP21 could bolster the therapeutic efficacy of TACE against HCC by instigating the ubiquitination and subsequent proteasomal degradation of PD‐L1. This dual‐action mechanism holds the promise of enhancing antitumour effects and circumventing resistance to TACE‐induced hypoxia and other pro‐survival signals. Through the present study, we endeavour to delineate the functional interplay between USP21 and PD‐L1 in the context of TACE treatment for hepatocellular carcinoma, thereby charting a novel combinatorial strategy aimed at improving clinical outcomes through the potentiation of tumour suppression and overcoming resistance mechanisms.

## 2 Methods

### 2.1 Cell Culture

The human HCC cell lines HepG2 were obtained from Cell Bank of the Chinese Academy of Sciences (Shanghai, China). Additional HCC cell lines Huh7 and MHCC97H were also used for validation experiments. HepG2 cells were cultured in Dulbecco's modified Eagle's medium (DMEM) (Gibco, CA, USA) supplemented with 10% FBS and 1% Penicillin/Streptomycin. Plasmids for PD‐L1 overexpression (PD‐L1‐pEX), empty vector (vector), lentiviral vector carrying USP21 shRNA (shUSP21#1: 5′‐GCAAGACCAUCUACAUCAATT‐3′; and a second independent shRNA shUSP21#2: 5′‐GCAAGACCAUCUACAUCAATT‐3′) and control vector (LV‐control, 5′‐UUCUCCGAACGUGUCACGUTT‐3′) were procured by Beyotime. Fluorescence analysis was implemented to assess the efficacy of transfection. PD‐L1 overexpression in HepG2 cells utilised 2 μg of cDNA, while USP21 expression was suppressed using LV‐USP21 shRNA. Lipofectamine2000 was employed for transfection following the manufacturer's protocol.

### 2.2 Constructed the HCC Mice Model and Dealt With Therapies

Two complementary mouse models were employed in this study. First, a diethylnitrosamine (DEN)‐induced orthotopic HCC model was established using pregnant C57BL/6 mice from the Laboratory Animal Center, Shanghai, China. All animal studies adhered to NIH guidelines. Male offspring aged 15 days were intraperitoneally injected with DEN (20 mg/kg) and subsequently administered TCPOBOP (3 mg/kg) every two weeks for 5 months to induce HCC. This model was used to evaluate the efficacy of TACE and its combination with USP21 knockdown, as TACE requires hepatic artery access. USP21 knockdown in this model was achieved by tail vein injection of adeno‐associated virus serotype 8 (AAV8) expressing USP21 shRNA (1 × 10^11^ viral genomes per mouse) four weeks after the final DEN injection. PD‐L1 overexpression was achieved by tail vein injection of AAV8‐PD‐L1 (1 × 10^11^ viral genomes per mouse).

Second, a subcutaneous xenograft model was used to assess the direct effects of USP21 knockdown and PD‐L1 overexpression on tumour growth without the influence of TACE. Male BALB/c nude mice (4–6 weeks old) received subcutaneous injections of 1 × 10^6^ HepG2 cells stably transfected with USP21 shRNA, PD‐L1 overexpression plasmid or appropriate controls into the right flank.

Mice underwent various treatments, including TACE therapy, subcutaneous injection of USP21 shRNA‐expressing cells into nude mice, combination therapy of TACE with USP21 shRNA, combination therapy of TACE with USP21 shRNA and PD‐L1 overexpression and a control group without treatment (*n* = 8 per group). For TACE therapy, the procedure was performed as described previously [16] with the following details: a PE‐10 polyethylene catheter was inserted into the hepatic artery of DEN‐induced HCC mice under isoflurane anaesthesia. A mixture of doxorubicin (2 mg/kg) and lipiodol (0.1 mL) was infused over 5 min to achieve chemoembolisation. Successful embolisation was confirmed by stagnation of blood flow and visible lipiodol deposition in the tumour region under a microscope. TACE was performed once, and mice were monitored for 21 days post‐treatment. Mice of USP21 shRNA treatment in the subcutaneous model received subcutaneous injections of HepG2 cells transfected with USP21 shRNA at approximately 1 × 10^6^ cells per injection site into the right flank. As for the reversal experiment, subcutaneous injections of cells overexpressing PD‐L1 along with USP21 shRNA were performed at the same dose. Mice in the control group underwent the same procedures but did not receive any therapeutic intervention. After 21 days of treatment, mice were anaesthetised with 2% isoflurane and hepatic cancer tissues or subcutaneous tumours were collected for analysis. Orthotopic HCC tissue volume was calculated using the formula: Vcm^3^ = 1/2 × LW^2^, where L and W represent the length and width of the tumours, respectively. For subcutaneous tumours, calliper measurements were taken every 3 days and volume was calculated using the same formula.

### 2.3 MTT Assay

Following cellular transfection, a 96‐well plate was used and 2 × 10^3^ cells were seeded into each well. The plate was then incubated for 24 h at 37°C to allow cell attachment and growth. After this incubation period, a 5 mg/mL MTT (3‐(4,5‐dimethylthiazol‐2‐yl)‐2,5‐diphenyltetrazolium bromide) solution was added to each well. The plate was returned to the incubator for an additional 4 h to allow viable cells to reduce the MTT to formazan crystals. After the incubation with MTT, the supernatants were carefully removed from each well. Subsequently, 150 μL of dimethyl sulfoxide (DMSO) was added to each well to dissolve the formazan crystals formed by metabolically active cells. The plate was gently shaken to ensure complete dissolution of the formazan. Cell viability was assessed by measuring the absorbance at 490 nm using a microplate reader. This process was repeated over a period of 6 days to monitor cell proliferation and viability.

### 2.4 Soft Agar Colony Formation Assay

Experiments were conducted using 24‐well plates. Where the base layer consisted of 0.8% purified agar mixed with complete growth medium, providing a solid support. The upper layer contained approximately 1 × 10^4^ cells per 250 μL suspended in 0.3% noble agar combined with complete medium, allowing for the growth of colonies. The plates were incubated at 37°C for 21 days to facilitate colony formation. After incubation, the cultures were stained with p‐iodonitrotetrazolium violet, which marks viable cell colonies. Finally, the colonies were imaged and counted using ImageJ software to assess the anchorage‐independent growth of the cells.

### 2.5 Cellular Motility Evaluation

Assessments were conducted in 24‐well transwell chambers. Cells were seeded into the upper compartment and allowed to migrate or invade towards the lower compartment for 12 or 24 h, respectively. Cells that migrated were subjected to Giemsa staining and enumerated by microscopic observation.

### 2.6 Immunohistochemistry for Ki67 Detection

Paraffin‐embedded and formalin‐fixed sections of HCC, alongside adjacent non‐tumour tissues, underwent deparaffinisation and rehydration. Endogenous peroxidase quenching was achieved by exposing tissue sections to a 3% hydrogen peroxide solution for a duration of 30 min. Antigen unmasking involved pressurised heating in the presence of sodium citrate buffer (pH 6) for 30 min. Samples were then incubated with a rabbit polyclonal anti‐Ki67 antibody (diluted 1:100, Thermo Fisher Scientific) overnight at 4°C. Following rinsing, sections were exposed to a secondary antibody (diluted 1:4000, Abcam) for 1 h, followed by staining with 3,3‐diaminobenzidine tetrahydrochloride (DAB). Haematoxylin served for counterstaining, and microscopic examination ensued. For negative controls, the primary antibody was substituted with PBS. Positive controls utilised human tonsil carcinoma tissue sections known to exhibit positive immunostaining.

### 2.7 Terminal Deoxynucleotidyl Transferase dUTP Nick End Labeling (TUNEL) Assay

For detecting apoptosis, cells were carefully seeded onto coverslips and fixation was performed by immersion in a 4% paraformaldehyde solution for 15 min at ambient temperature. Subsequently, 0.1% Triton X‐100 in PBS was added and then incubated with TUNEL reaction mixture (containing TdT enzyme and fluorescein‐labelled dUTP) in a humidified chamber at 37°C for 1 h. After washing with PBS, cells were counterstained with 4′,6‐diamidino‐2‐phenylindole (DAPI) for nuclear staining. This method enabled the visualisation and quantification of apoptotic cells through fluorescence microscopy or high‐content imaging systems.

### 2.8 Flow Cytometry Analysis

Apoptosis levels were quantitatively assessed by flow cytometry analysis. Following trypsinisation, the cells were washed with phosphate‐buffered saline (PBS) and resuspended in an Annexin V binding buffer. The cell suspension was subsequently treated with Annexin V‐fluorescein isothiocyanate (FITC) and propidium iodide (PI) at the concentrations specified by the manufacturer's instructions. This mixture was incubated for 15 min at ambient temperature in the absence of light. This procedure enabled the differentiation between early apoptotic cells (Annexin V‐positive, PI‐negative) and late apoptotic/necrotic cells (Annexin V‐positive, PI‐positive).

### 2.9 Quantitative Polymerase Chain Reaction (qRT‐PCR)

Total RNA was isolated from treated cells using TRIzol reagent, per manufacturer's instructions. RNA was quantified, and 1 μg was reverse transcribed to cDNA. qPCR was performed with gene‐specific primers and SYBR Green mix. GAPDH served as the normalisation control. Relative USP21 and PD‐L1 expression was calculated by the 2^−ΔΔCt^ method. Primer sequences:

USP21:5′‐ACTGGGGATACGATGGCTGA‐3′ (sense);

5′‐ACAGGCTGGACCCACAATC‐3′ (antisense).

PD‐L1:5′‐TAT GGTGGTGCCGACTACAA‐3′ (sense);

5′‐TGCTTGTCCAGATGACTT CG‐3′ (antisense).

GAPDH: 5′‐GCACCGTCAAGGCTGAGAAC‐3′ (sense);

5′‐TGGTGAAGACGCCAGTGGA‐3′ (antisense).

### 2.10 Immunoblotting

Protein expression was evaluated by western blotting. Cells were lysed in RIPA buffer with inhibitors. Lysates were centrifuged, and supernatant protein concentrations determined by BCA assay. Equal protein amounts were separated by SDS‐PAGE, transferred to PVDF membranes and blocked. Membranes were incubated with primary antibodies overnight at 4°C, followed by HRP‐conjugated secondary antibodies. Protein bands were visualised by ECL and quantified using ImageJ. The following antibodies were used: Anti‐PD‐L1 (#ab213524, Abcam, 1:1000); anti‐USP21, human (#sc‐79305, Santa Cruz Biotechnology, 1:200); anti‐USP21, mouse (#ab171028, Abcam, 1:500); anti‐Flag‐HRP (#F1804, Sigma, 1:10,000), anti‐HA (#901501, BioLegnd, WB 1:10,000); anti‐Ub (P4D1) (#8240, CST, 1:100); anti‐K48 (#05‐1307, Milipore, 1:500); anti‐TGFβR2 (#BS7266, Bioworld, 1:1000); anti‐PTPN14 (#13808S, CST, 1:1000); anti‐β‐actin (#A2228, Sigma, 1:5000); Anti‐mouse IgG, HRP‐linked (#7076s, CST, 1:10,000); goat anti‐mouse IgG (H+L) secondary antibody, HRP‐linked (#31430, Thermo Fisher Scientific, 1:10,000); Rabbit anti‐Goat IgG (H+L) secondary antibody (#61‐1620, Thermo Fisher Scientific, 1:10,000).

### 2.11 Immunoprecipitations

Following the methodology previously described [17], plasmids encoding PD‐L1 and USP21 were directly constructed via Beyotime (shanghai, China). Subsequent to transfection of these plasmids into HepG2 cells, the post‐transfection cell population was collected and subjected to lysis using RIPA buffer fortified with protease inhibitor compounds. Followed by incubation at 4°C for 30 min. Upon centrifugation, cell debris was removed and the supernatant was collected. Immunoprecipitation was then carried out by incubating the supernatant with anti‐PD‐L1 antibody or control IgG, followed by the addition of protein A/G agarose beads. The mixture was gently rotated overnight at 4°C, and after incubation, the beads were washed four times with RIPA buffer to eliminate nonspecific binding. Immunoprecipitated proteins were eluted from the beads, boiled in SDS sample buffer for 10 min and separated by SDS‐PAGE following the immunoblotting procedure.

### 2.12 In Vitro Deubiquitination Assay

HepG2 cells were transfected with Flag‐PD‐L1, HA (hemagglutinin‐tagged)‐USP21‐WT and HA‐USP21‐CA (catalytically inactive mutant) expression plasmids. Following a 48‐h transfection period, cells were treated with MG132 (20 μM) for 6 h to facilitate protein stabilisation before collection. Cell lysates were prepared using RIPA lysis buffer supplemented with protease inhibitors and then centrifuged at 15,000 *g* for 10 min to obtain the protein‐enriched supernatant. Subsequently, the supernatant was incubated with Flag/HA‐beads for 6 h to facilitate the selective pull‐down of PD‐L1 and USP21 proteins. PD‐L1 and USP21 proteins were meticulously purified from the cell extracts utilising the Flag/HA‐beads in lysis buffer. For the deubiquitination assay, ubiquitinated PD‐L1 underwent incubation with either HA‐USP21‐WT or HA‐USP21‐CA in DUB Buffer (composed of 50 mM Tris pH 7.5, 50 mM NaCl, 5 mM DTT, 5 mM MgCl_2_) at 30°C on a rotary shaker for a precise 2‐h duration. Following this incubation period, proteins were effectively eluted from the beads and subjected to a brief heating step at 100°C for 10 min. The ubiquitination status of PD‐L1 was meticulously evaluated via immunoblotting, adhering to standardised protocols.

### 2.13 Cycloheximide Chase Assay and Densitometric Analysis

To assess PD‐L1 protein stability, HepG2 cells transfected with vector or HA‐USP21 were treated with cycloheximide (CHX, 50 μg/mL) for 0, 2, 4, 6 and 8 h. Cells were harvested at each time point, and lysates were subjected to western blotting with anti‐PD‐L1 and anti‐β‐actin antibodies. Protein bands were quantified using ImageJ software, and PD‐L1 signals were normalised to β‐actin. The normalised values were plotted against time, and the half‐life was estimated by fitting an exponential decay curve. Three independent experiments were performed, and data are presented as mean ± standard deviation (SD).

### 2.14 Statistical Analysis

Prism 8.0 was used to examine results. The data was displayed as mean ± SD based on at least three different experiments. Student's *t*‐test was used to evaluate group comparisons for two groups, and one‐way ANOVA with Tukey's post hoc test for multiple comparisons. At least three replications of each experiment were conducted. *p* values < 0.05 were regarded as statistically significant.

## 3 Results

### 3.1 Silencing USP21 Attenuates Proliferation and Invasion of HepG2 Cells

**FIGURE 1:**
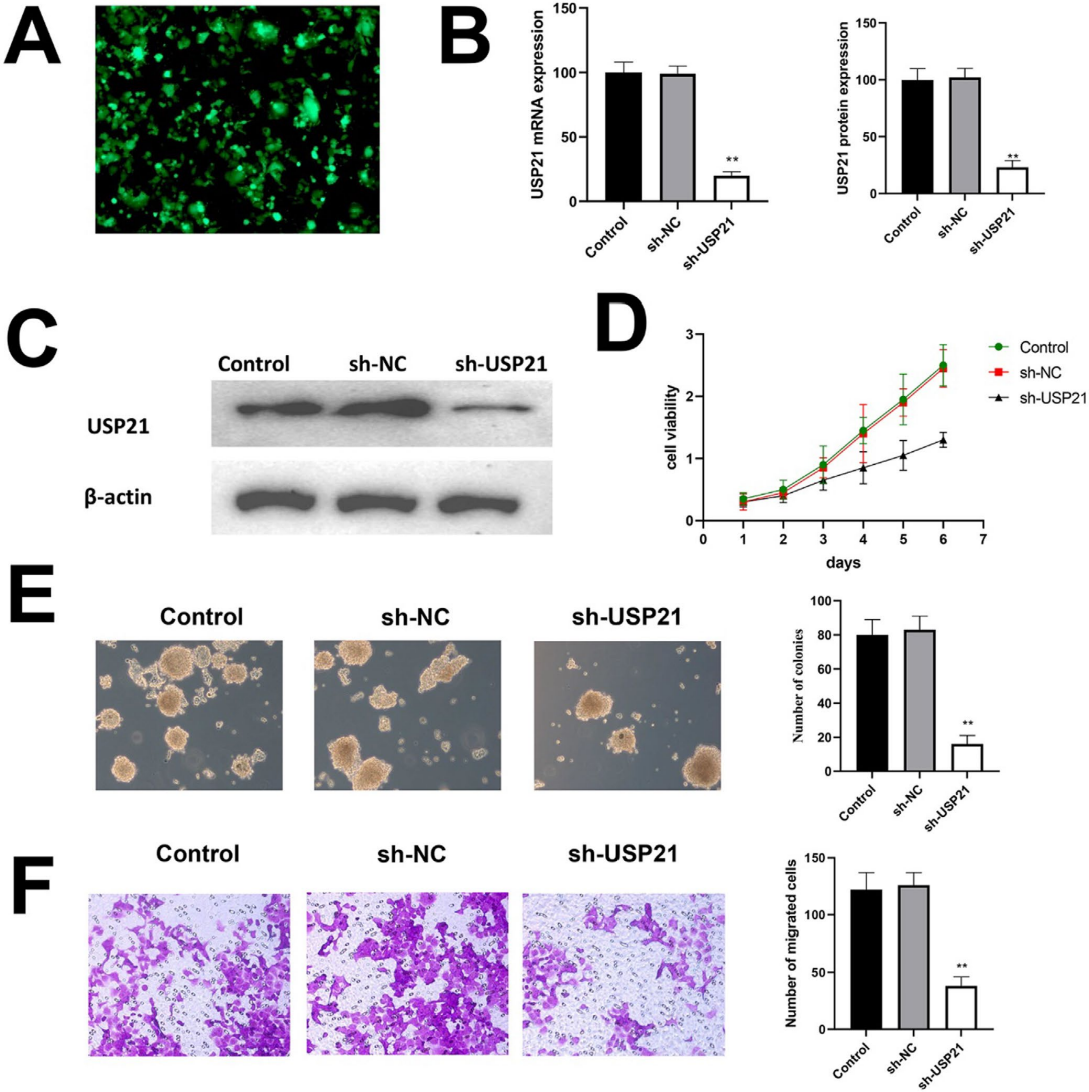
Knockdown of USP21 expression in HepG2 cells. (A) Fluorescence microscopy showing GFP expression after lentiviral transduction of USP21 shRNA. (B, C) qRT‐PCR and Western blot analysis of USP21 mRNA and protein levels. (D) MTT assay for cell proliferation. (E) Soft agar colony formation assay. (F) Transwell migration assay demonstrating decreased invasive ability of HepG2 cells upon USP21 silencing. Scale bar = 50 μm. **p < 0.01.

To explore the functional role of USP21 in hepatocellular carcinoma (HCC), we first knocked down USP21 expression in HepG2 cells using a lentiviral vector carrying USP21‐specific shRNA (sh‐USP21). Fluorescence microscopy confirmed the successful transduction of HepG2 cells with the sh‐USP21 lentivirus, as evidenced by the expression of green fluorescent protein (GFP) (Figure 1A). Subsequent qRT‐PCR and Western blot analyses demonstrated a significant reduction in both mRNA and protein levels of USP21 in the sh‐USP21‐transfected cells, compared to the control group (Figure 1B,C), indicating the successful establishment of the USP21 knockdown model. The functional consequences of USP21 silencing were then evaluated. MTT assays revealed that USP21 knockdown significantly inhibited the proliferation of HepG2 cells (Figure 1D). Moreover, the soft agar colony formation assay showed that the anchorage‐independent growth capacity of HepG2 cells was markedly suppressed upon USP21 silencing (Figure 1E). Additionally, the transwell migration assay demonstrated that the invasive ability of HepG2 cells was impaired by the knockdown of USP21 (Figure 1F). Similar results were obtained with a second independent shRNA (shUSP21#2) and in Huh7 cells (data not shown), confirming the specificity and generalisability of the effects. These results suggested that silencing the expression of USP21 could effectively attenuate the proliferative and invasive capabilities of HCC cells, underscoring the potential of targeting USP21 as a therapeutic strategy.

### 3.2 Silencing USP21 Enhances the Therapeutic Efficacy of TACE Against HCC in Orthotopic Mice

**FIGURE 2:**
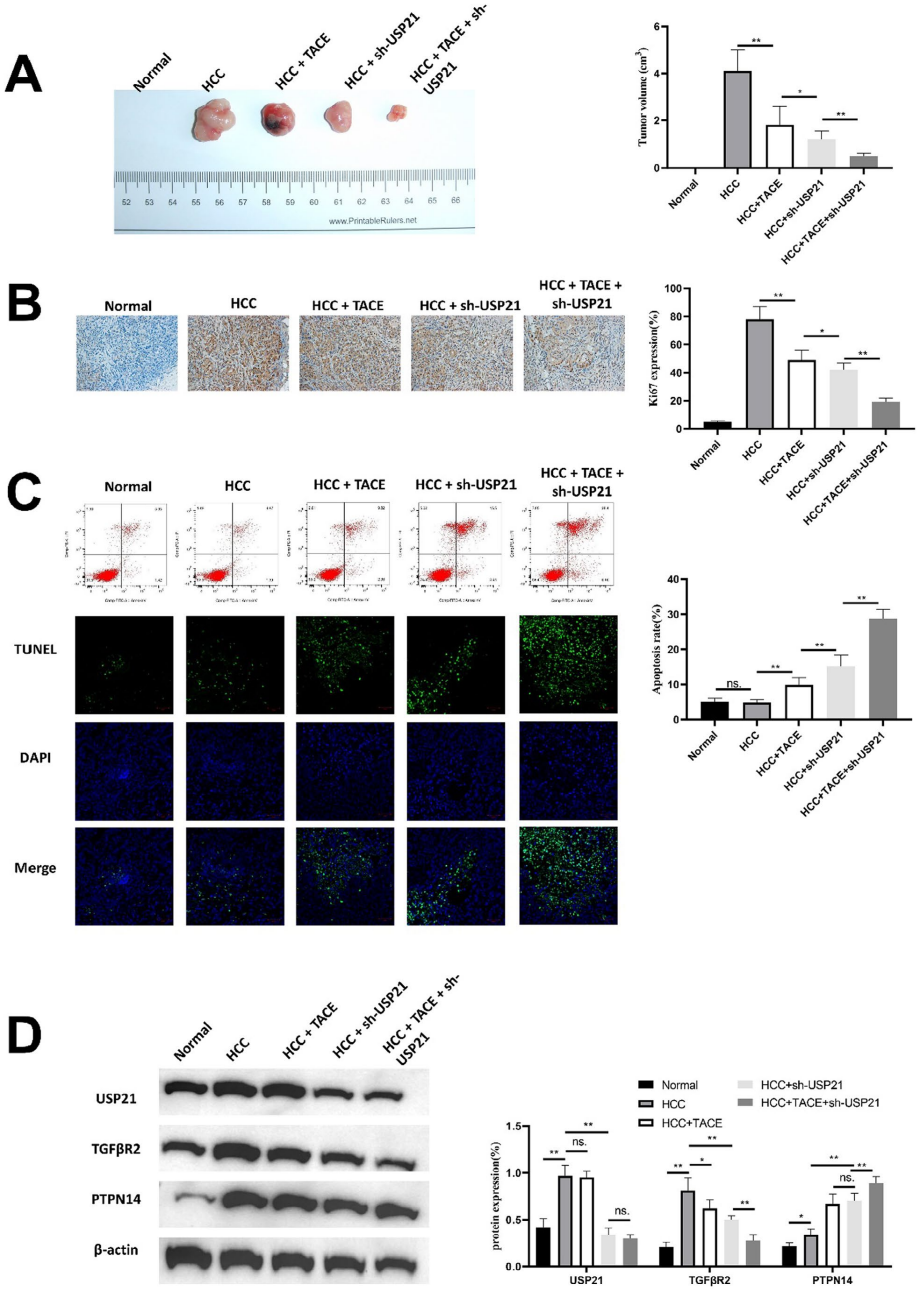
Silencing USP21 enhances the therapeutic efficacy of TACE against HCC in mice. (A) Tumour volume. (B) Immunohistochemistry for Ki‐67. (C) Flow cytometry analysis and TUNEL assay for apoptosis. (D) Western blot analysis of TGF‐βR2 and PTPN14 expression. *n* = 8 mice per group. Scale bar = 50 μm. *p < 0.05; **p < 0.01; ns, not significant.

To evaluate the impact of USP21 knockdown on the therapeutic response to transarterial chemoembolisation (TACE) in hepatocellular carcinoma (HCC), we established a DEN‐induced HCC mouse model and subjected the animals to various treatments. USP21 knockdown was achieved by tail vein injection of AAV8‐shUSP21, and TACE was performed as described in Methods. As shown in Figure 2A, TACE treatment or USP21 knockdown alone significantly reduced tumour volume compared to the control group. Importantly, the combination of TACE and USP21 silencing yielded the most pronounced inhibitory effect on tumour growth. Immunohistochemical analysis of the proliferation marker Ki‐67 revealed that HCC tissues exhibited markedly higher Ki‐67 expression compared to normal liver tissues (Figure 2B). Both TACE treatment and USP21 knockdown effectively suppressed Ki‐67 levels, and the combined therapy exhibited the greatest reduction in Ki‐67 expression. The TUNEL assay was employed to assess apoptosis in the HCC tissues. As shown in Figure 2C, TACE and USP21 knockdown significantly enhanced apoptosis, and the combination of the two treatments further increased the apoptotic rate. Moreover, Western blot analysis demonstrated that TACE and USP21 silencing independently decreased the expression of TGF‐βR2, a pro‐tumourigenic receptor, while increasing the levels of PTPN14, a tumour suppressor (Figure 2D). The combinatorial therapy exhibited the most pronounced effects on these molecular markers. Collectively, above findings indicated that silencing USP21 might potentiate the therapeutic efficacy of TACE against HCC, suggesting a promising strategy for improving clinical outcomes.

### 3.3 USP21 Stabilises PD‐L1 Through Deubiquitination

**FIGURE 3:**
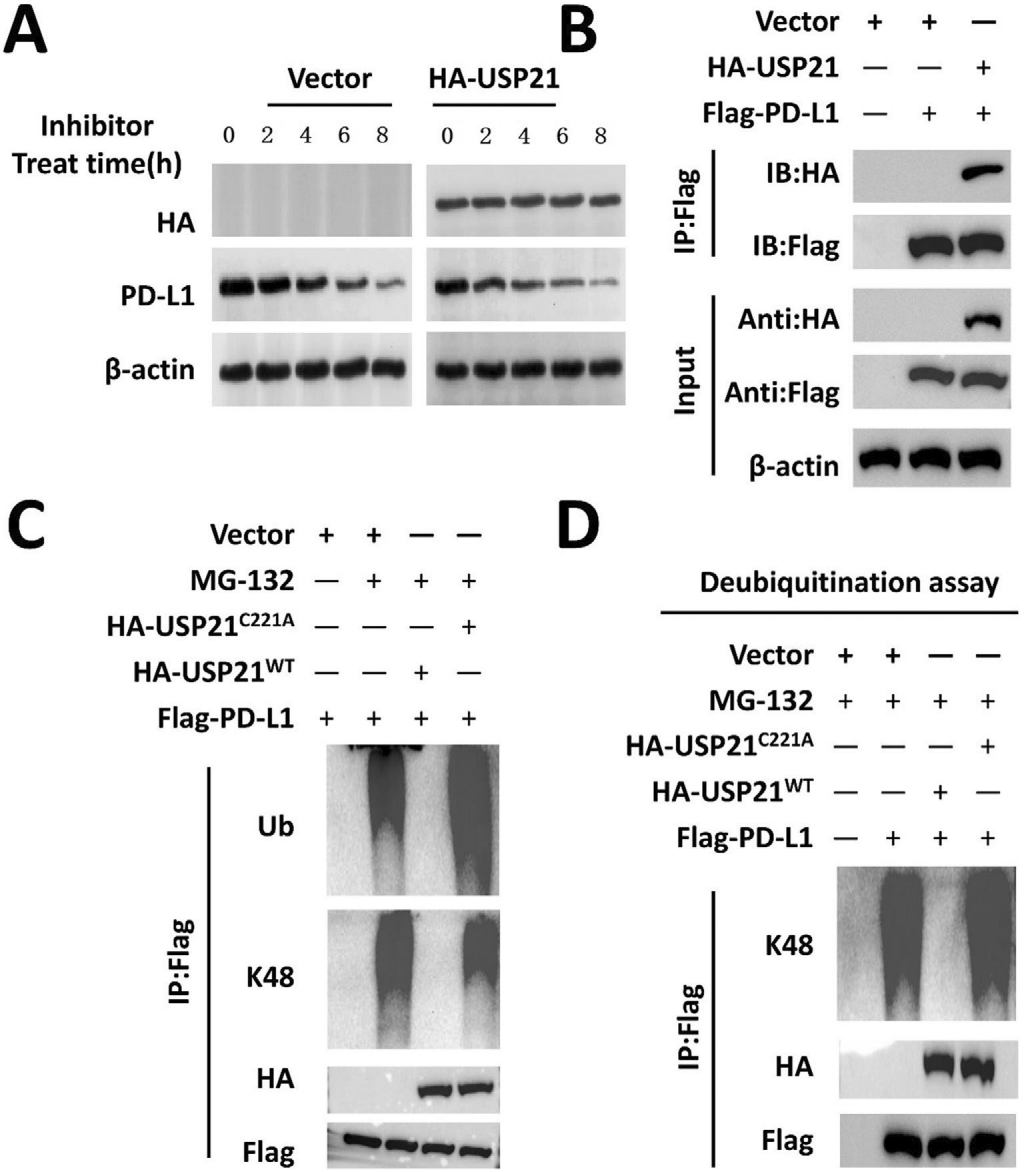
USP21 stabilises PD‐L1 through deubiquitination. (A) Western blot analysis showing the effect of USP21 overexpression on PD‐L1 protein levels. (B) Co‐immunoprecipitation (co‐IP) experiment confirming the interaction between USP21 and PD‐L1. (C) Ubiquitination assay demonstrating the ability of USP21 to suppress MG132‐induced PD‐L1 polyubiquitination. (D) In vitro deubiquitination assay revealing that wild‐type USP21, but not the catalytically inactive mutant USP21‐CA, can remove polyubiquitin chains from PD‐L1.

Previous studies have reported that the deubiquitinase USP21 could interact with and stabilise PD‐L1 [17]. To further elucidate this regulatory mechanism in HCC, we first overexpressed USP21 in HepG2 cells and observed a marked reduction in PD‐L1 protein turnover, suggesting that USP21 is involved in maintaining PD‐L1 stability (Figure 3A). To quantitatively assess this effect, we performed cycloheximide (CHX) chase assays with multiple time points. Densitometric analysis of PD‐L1 bands normalised to β‐actin from three independent experiments demonstrated that USP21 overexpression significantly slowed PD‐L1 degradation compared to vector control (Figure 3A and Table 1). The estimated half‐life of PD‐L1 increased from approximately 3.5 h in control cells to > 8 h in USP21‐overexpressing cells (*p* < 0.01). Co‐immunoprecipitation (co‐IP) experiments confirmed the direct physical interaction between USP21 and PD‐L1 (Figure 3B). It was well established that protein degradation is often accompanied by K48‐linked polyubiquitination. Therefore, we examined the ubiquitination status of PD‐L1 in the presence of the proteasome inhibitor MG132. We found that overexpression of wild‐type USP21 (HA‐USP21‐WT) abolished the MG132‐induced polyubiquitination of PD‐L1, whereas the catalytically inactive mutant USP21‐C221A (HA‐USP21‐C221A) failed to do so, leading to the accumulation of polyubiquitinated PD‐L1 (Figure 3C). To directly assess the deubiquitinating activity of USP21 on PD‐L1, we performed an in vitro deubiquitination assay. Purified PD‐L1 proteins were incubated with either HA‐USP21‐WT or HA‐USP21‐C221A for 2 h. As shown in Figure 3D, while PD‐L1 was extensively ubiquitinated, USP21‐WT significantly reduced the levels of polyubiquitinated PD‐L1, whereas the catalytically inactive USP21‐C221A mutant did not exhibit this deubiquitinating activity. The data demonstrated that USP21 directly interacted with and deubiquitinate PD‐L1, thereby enhancing its stability and promoting its accumulation in HCC cells.

**TABLE 1:** Densitometric quantification of PD‐L1 protein levels relative to β‐Actin in CHX chase assays.

Time (h)	Vector (mean ± SD, *n* = 3)	HA‐USP21 (mean ± SD, *n* = 3)	*p*
0	1.00 ± 0.05	1.00 ± 0.04	> 0.05
2	0.72 ± 0.06	0.91 ± 0.05	< 0.05
4	0.51 ± 0.04	0.83 ± 0.06	< 0.01
6	0.34 ± 0.03	0.76 ± 0.05	< 0.001
8	0.21 ± 0.02	0.68 ± 0.04	< 0.001

### 3.4 Overexpression of PD‐L1 Reverses the Inhibitory Effects of USP21 Knockdown on HepG2 Cell Proliferation and Invasion

**FIGURE 4:**
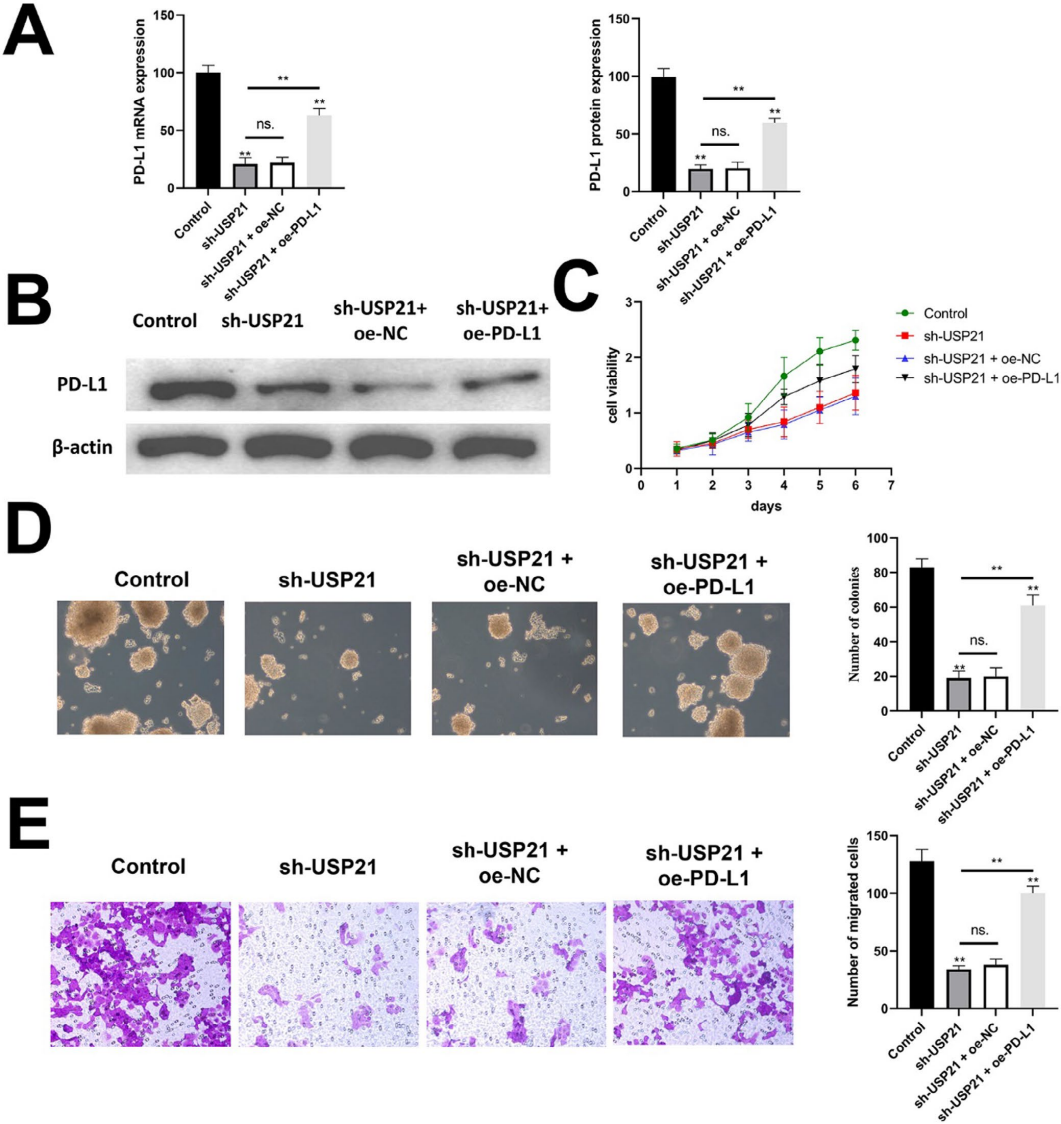
Overexpression of PD‐L1 reverses the inhibitory effects of USP21 knockdown on HepG2 cell proliferation and invasion. (A, B) qRT‐PCR and Western blot analysis showing the downregulation of PD‐L1 expression upon USP21 silencing, which was restored by PD‐L1 overexpression. (C) MTT assay for cell proliferation. (D) Soft agar colony formation assay. (E) Transwell invasion assay. Scale bar = 50 μm. **p < 0.01; ns, not significant.

To further investigate the involvement of PD‐L1 in the functional consequences of USP21 silencing, we knocked down USP21 expression in HepG2 cells and observed a significant reduction in both PD‐L1 mRNA and protein levels (Figure 4A,B). Importantly, ectopic overexpression of PD‐L1 in these USP21‐depleted cells restored the PD‐L1 expression to levels comparable to the control group. The functional assays revealed that the inhibitory effects of USP21 knockdown on cell proliferation (Figure 4C), anchorage‐independent growth (Figure 4D), and invasion (Figure 4E) were all effectively reversed by the overexpression of PD‐L1. These results suggested that the tumour‐suppressive effects induced by USP21 silencing were largely mediated through the downregulation of PD‐L1, indicating that USP21 exerted its pro‐tumourigenic functions in HCC, at least in part, by stabilising the PD‐L1 oncoprotein. Targeting this USP21/PD‐L1 axis might represent a promising therapeutic strategy for HCC management.

### 3.5 Overexpression of PD‐L1 Reverses the Enhanced Therapeutic Efficacy of TACE in the Presence of USP21 Knockdown

**FIGURE 5:**
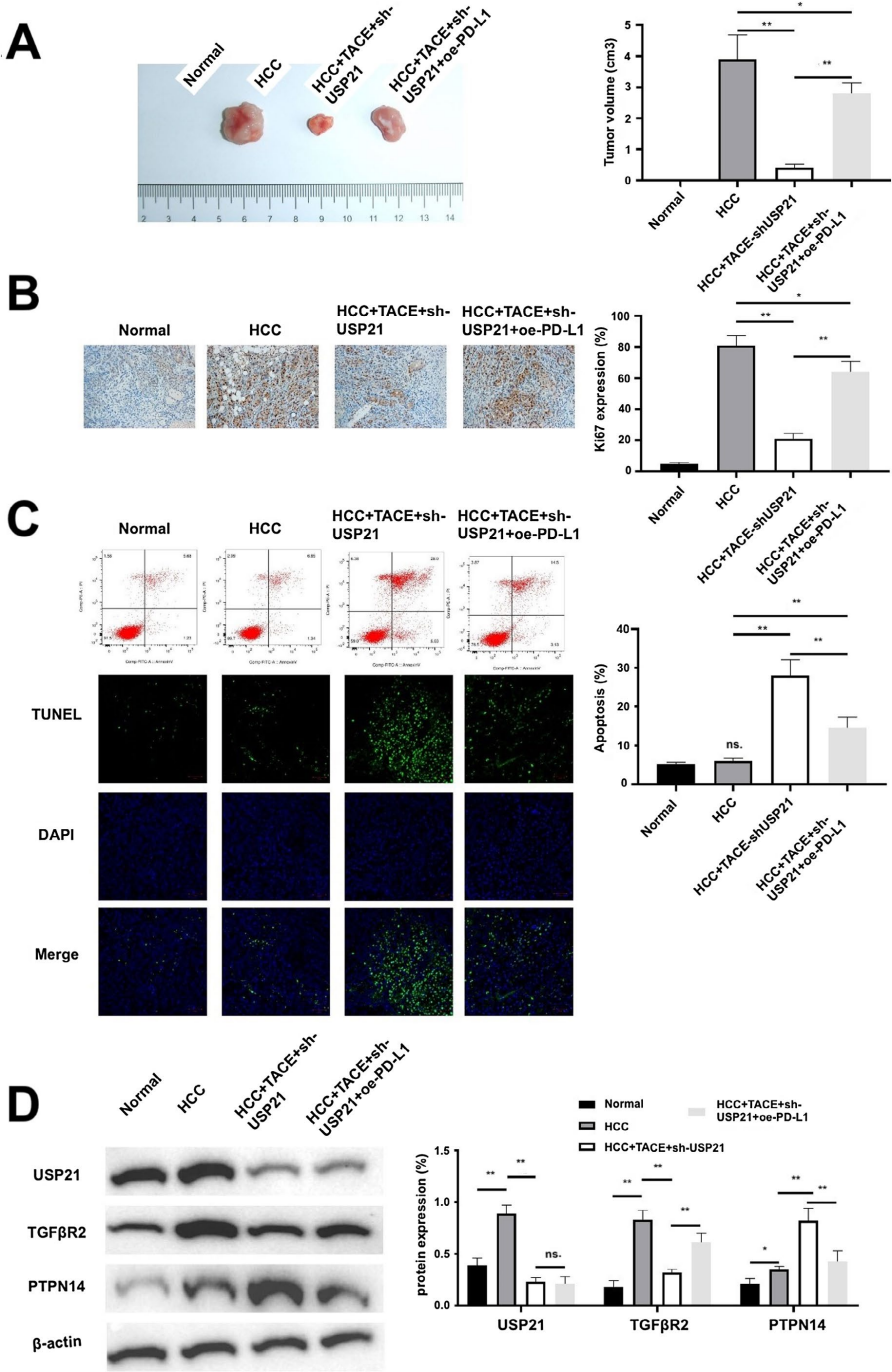
Overexpression of PD‐L1 reverses the enhanced therapeutic efficacy of TACE in the presence of USP21 knockdown. (A) Tumour volume. (B) Immunohistochemistry for Ki‐67. (C) Flow cytometry analysis and TUNEL assay for apoptosis. (D) Western blot analysis of TGF‐βR2, PTPN14 and USP21 expression. *n* = 8 mice per group. Scale bar = 50 μm. *p < 0.05; **p < 0.01; ns, not significant.

To determine whether the antitumour effects of the combination of TACE and USP21 silencing were mediated through the regulation of PD‐L1, we conducted a rescue experiment by overexpressing PD‐L1 in the orthotopic HCC mouse model using AAV8‐PD‐L1. As shown in Figure 5A, the inhibitory effect of the TACE plus USP21 shRNA treatment on tumour growth was significantly attenuated by the overexpression of PD‐L1. Similarly, the reduced Ki‐67 expression induced by the combined therapy was rescued by PD‐L1 overexpression (Figure 5B). The TUNEL assay revealed that the enhanced apoptosis observed in the TACE plus USP21 shRNA group was also reversed by PD‐L1 overexpression (Figure 5C). Furthermore, Western blot analysis demonstrated that the downregulation of the pro‐tumourigenic TGF‐βR2 and the upregulation of the tumour suppressor PTPN14 mediated by the combined treatment were both abrogated upon PD‐L1 overexpression, while USP21 levels remained unchanged (Figure 5D). These results indicated that the enhanced therapeutic efficacy of TACE in the presence of USP21 knockdown was largely dependent on the regulation of PD‐L1. Overexpression of PD‐L1 effectively reversed the antitumour effects induced by the combined TACE and USP21 silencing therapy, underscoring the central role of the USP21/PD‐L1 axis in HCC progression and treatment response.

## 4 Discussion

PD‐L1 overexpression in HCC has been consistently associated with tumour aggressiveness and unfavourable clinical outcomes [18, 19]. Targeting PD‐L1 has been a common immunotherapy approach for HCC treatment [20]. Beyond genomic alterations and epigenetic modifications, emerging evidence suggests that post‐translational mechanisms, such as deubiquitination, play a crucial role in regulating PD‐L1 stability and expression [21, 22]. Similarly, in the present study, we elucidated that the deubiquitinase USP21 stabilised PD‐L1 through deubiquitination, preventing its proteasomal degradation and promoting the progression of HCC. This finding reveals intricate post‐translational regulation of PD‐L1 in HCC and provides a potential therapeutic target for modulating its expression.

The multifaceted roles of USP21 in cancer pathogenesis have been extensively documented, with its aberrant expression contributing to hallmarks such as uncontrolled proliferation, invasion and chemoresistance [23, 24]. In HCC specifically, upregulated USP21 levels have been consistently linked to cell proliferation, anchorage‐independent growth and cell cycle progression by stabilising MEK2 [25]. Regarding clinical aspects, it was reported that the upregulated USP21 expression was positively related to shorter survival in HCC [26]. Moreover, recent studies in other cancer types have unveiled USP21's ability to interact with and deubiquitinate PD‐L1, thereby stabilising its expression [17]. Complementing these findings, our study corroborates this regulatory mechanism in the context of HCC, wherein USP21 directly binds and deubiquitinates PD‐L1, leading to increased stability and accumulation of this immune checkpoint protein.

Although TACE has long been the standard of care for unresectable HCC, its efficacy remains compromised by inevitable tumour recurrence and metastasis, partially attributed to the induction of hypoxia and subsequent upregulation of PD‐L1, fostering an immunosuppressive tumour microenvironment [27]. To overcome these limitations, recent studies have explored combinatorial strategies to potentiate the effects of TACE, including the addition of immune checkpoint inhibitors targeting the PD‐1/PD‐L1 axis [16]. Complementing these efforts, our study investigated a novel approach by silencing USP21 in conjunction with TACE treatment. Remarkably, this dual‐action approach significantly enhanced the therapeutic efficacy of TACE against HCC in a preclinical mouse model, suggesting that targeting the USP21/PD‐L1 axis could potentiate the antitumour effects of TACE by modulating PD‐L1 levels and overcoming resistance mechanisms.

Interestingly, we observed that USP21 knockdown also led to a decrease in PD‐L1 mRNA levels, suggesting that USP21 may influence PD‐L1 expression at the transcriptional level or through mRNA stability, in addition to its well‐established role in deubiquitination. This could be mediated by indirect effects on transcription factors or signalling pathways that regulate PD‐L1 transcription. Nevertheless, our CHX chase and ubiquitination assays provide direct evidence that USP21 primarily stabilises PD‐L1 protein by removing polyubiquitin chains, highlighting post‐translational regulation as a key mechanism.

In summary, our study unveiled a pivotal role for the deubiquitinase USP21 in stabilising PD‐L1 expression in HCC, thereby promoting tumour progression and therapy resistance. Silencing USP21 effectively attenuated HCC cell proliferation and invasion, while enhancing the therapeutic efficacy of TACE in a preclinical mouse model. Notably, the potentiated antitumour effects of the TACE/USP21 knockdown combination were largely mediated through the downregulation of PD‐L1, as demonstrated by rescue experiments involving PD‐L1 overexpression. These findings underscore the USP21/PD‐L1 axis as a promising therapeutic target in HCC and suggest that combining TACE with USP21 inhibition could represent a novel strategy to overcome resistance mechanisms and improve clinical outcomes. However, it is important to acknowledge several limitations of our study. First, future studies are needed to validate the USP21/PD‐L1 axis in diverse HCC subtypes and potentially other malignancies. Such work would clarify whether USP21 knockdown exhibits similar therapeutic benefits across a broader range of tumour types, expanding the clinical relevance of targeting USP21. In this study, we have extended our key findings to Huh7 cells and used a second independent shRNA to minimise off‐target effects, but further validation in patient‐derived models is warranted. Second, while our CHX chase assays with densitometric quantification now provide robust evidence for PD‐L1 protein stabilisation by USP21, future studies should incorporate pulse‐chase labeling experiments to quantitatively assess the dynamics of PD‐L1 stability and turnover with USP21 overexpression, providing even more definitive evidence for this mechanism. Another limitation of our study is the use of AAV‐mediated gene delivery in vivo, which may not fully recapitulate the effects of pharmacological USP21 inhibition. Future research should address these limitations by incorporating a broader range of HCC cell lines and patient‐derived xenograft models to validate our findings. Investigating the potential off‐target effects and developing more specific USP21 inhibitors will be crucial for advancing this therapeutic strategy. Moreover, clinical studies are needed to explore the translational potential of targeting the USP21/PD‐L1 axis in HCC treatment.

## Author Contributions

**Yun Tao:** conceptualization, investigation, funding acquisition, writing – original draft, writing – review and editing, project administration, formal analysis, validation, visualization, methodology, resources, supervision, software. **Wenhui Yu:** writing – review and editing, visualization, validation, formal analysis, resources, supervision. **Wenge Yang:** writing – review and editing, validation, visualization, formal analysis. **Jie Li:** writing – review and editing, visualization, validation, formal analysis, funding acquisition. **Qinghua Wu:** writing – review and editing, visualization, validation, methodology, project administration, formal analysis.

## Funding

Health Commission Surface Project of Wuxi City, Jiangsu Province (Grant M202321); Clinical Research and Translational Medicine Research Project of The Affiliated Hospital of Jiangnan University (Grant LCYJ202324).

## Ethics Statement

Ethical approval was given by The Affiliated Hospital of Jiangnan University.

## Conflicts of Interest

The authors declare no conflicts of interest.

## Data Availability

The data that support the findings of this study are available from the corresponding author upon reasonable request.
